# Effects of oral cysteine leukotriene receptor antagonist-montelukast on adenoid lymphoid tissue: a histopathological study under light microscope

**DOI:** 10.3389/fphar.2023.1285647

**Published:** 2023-11-02

**Authors:** Zhengying Wang, Xiuling Wu, Jinghong Liu, Yu Wang, Yue Zhang, Yan Wu, Yingxue Kang, Ronghai Zhang, Jin Li, Delong Liu

**Affiliations:** ^1^ Department of Otorhinolaryngology Head and Neck Surgery, Dalian municipal Central Hospital, Dalian, Liaoning, China; ^2^ China Medical University, Shenyang, Liaoning, China; ^3^ Dalian Medical University, Dalian, Liaoning, China; ^4^ Department of Otorhinolaryngology, Panjin Municipal Central Hospital, Panjin, Liaoning, China

**Keywords:** adenoid hypertrophy, cysteine leukotriene receptor antagonist, montelukast, histopathological, lymphocytes

## Abstract

**Background:** Adenoidal hypertrophy (AH) is one of the most common causes of upper airway obstruction in children. Drug and surgical treatment are the typical treatment of AH. The study on the inflammatory mechanism of AH in children provides a new idea for preoperative intervention and non-surgical treatment with anti-inflammatory drugs such as montelukast sodium (a cysteine leukotriene receptor antagonist). The aim of this study is to evaluate the effect of montelukast sodium on adenoidal lymphoid tissue pathology in children with AH under light microscope.

**Objective:** To study whether there is any change in pathology of the adenoidal lymphoid tissue under the light microscope compared with the control group in children with moderate to severe simple AH treated with montelukast sodium for 1 month before operation.

**Materials and methods:** Twenty patients (8 males, 12 females, 3–8 years old) with moderate to severe AH who were prepared for surgical treatment were selected. All the patients were examined by Nasopharyngeal CT and hemocyte analysis before operation. 20 subjects were randomly divided into two groups: One group was given montelukast chewable tablets 5 mg/d, qn, for 4 weeks; The control group was given placebo 5 mg/d, qn, for 4 weeks. After 4 weeks, the adenoids were removed and examined histopathology.

**Results:** Compared with the control group, the number of lymphocytes in the blood cell analysis of the study group was significantly reduced, with a statistically significant difference (*p* < 0.05). And the number of germinal centers in adenoid tissue of the study group was relatively reduced, no small cyst was found in the epithelium, and the degree of inflammatory cell infiltration was reduced, with a statistically significant difference (*p* < 0.05).

**Conclusion:** Montelukast can reduce the number of reactive cells, the number of lymphocytes in blood cells and blood vessels in adenoid lymphoid tissue, which can provide a new idea for preoperative intervention and non-surgical treatment of adenoid hypertrophy in children. However, this is only a pilot study and a longer treatment period is needed to assess the long-term effects of montelukast sodium on adenoid lymphoid tissue.

**Clinical Trial Registration**: www.Chictr.org.cn, identifier ChiCTR2300075040.

## 1 Introduction

The adenoids are lymphoepithelial organs located at the top of the nasopharynx. The lymphoid tissue consists of lymphoid follicles, germinal centers, and interfollicular areas (Bykova and Satdykova, 2002). It usually has the ability to fight respiratory infections. There are 12–15 shallow crypts, covered by columnar ciliated airway epithelium, and numerous goblet cells. Normal adenoids reach their maximum size between the ages of 3 and 7 years, and if infection does not occur, the adenoidal tissue may degenerate over time (Di et al., 2016). However, under the repeated stimulation of chronic inflammation, adenoids undergo pathological hyperplasia, causing adenoidal hypertrophy (AH). Untreated adenoidal hypertrophy can lead to nasal congestion, snoring, obstructive sleep apnea (OSA), oral breathing, ear and sinus infections, growth retardation and craniofacial abnormalities, and reduced quality of life (Bhargava and Chakravarti, 2014; Shokouhi et al., 2015).

Adenoid hypertrophy is mainly treated with medical therapy and surgery. For children with severe adenoid hypertrophy, the first choice of treatment is surgical resection (Kuhle et al., 2020). Given the perioperative risks and estimated recurrence rate of up to 20% postoperatively, there has been a growing interest in minimally invasive alternatives to adenoidectomy recently. In recent years, the study of the inflammatory mechanism of AH in children has provided new ideas for the clinical use of anti-inflammatory drugs for preoperative intervention and non-surgical treatment. Children with AH develop systemic inflammation represented by an increase in C-reactive protein (Gozal et al., 2007; Li et al., 2008). Overexpression of human cysteinyl-leukotriene receptor-1 in enlarged adenoid tissue, which interacts with leukotrienes and mediates the occurrence of inflammation. Therefore, anti-inflammatory agents with safe therapeutic properties may become a method of preoperative intervention or alternative treatment for adenoidectomy surgery (Goldbart et al., 2004; Kaditis et al., 2008). As a cysteine leukotriene receptor antagonist, montelukast sodium is effective, safe, well tolerated, and has been approved by the US Food and Drug Administration (FDA). According to research, montelukast sodium can act on adenoid lymphoid tissue, reducing its reactive inflammatory changes and possibly reducing its size (Goldbart et al., 2012). Due to the fact that enlarged adenoids are composed of enlarged lymphoid tissue, anti-inflammatory drugs are considered a potential non-surgical treatment option for children with adenoid hypertrophy (Kuhle et al., 2020). So far, there have been no studies on the histological effects of montelukast sodium on adenoid tissue.

The purpose of this randomized, double-blind, placebo-controlled study is to examine the effect of montelukast sodium on adenoid histopathology in children with AH under light microscope after 4 weeks of oral administration.

### 1.1 Materials and methods

#### 1.1.2 Study sample

This double-blind, randomized, placebo-controlled prospective study was conducted from January 2021 to August 2021 at the Department of Otolaryngology, Head and Neck Surgery, Dalian municipal Central Hospital. The research plan was reviewed and approved by the Ethics Committee of the Affiliated Central Hospital of Dalian University of Technology, and was approved by the child’s parents/legal guardians before the study entered. If they are children over 6 years old, consent was also obtained. And signed an informed consent form. All patients were hospitalized for surgery and no postoperative complications occurred.

We included a total of 20 research subjects. Inclusion criteria: 1) Age 3–8 years; 2) Nasal endoscopy and nasopharyngeal CT examination were performed in the Department of Otolaryngology, Head and Neck Surgery, Affiliated Central Hospital of Dalian University of Technology. Hypertrophic adenoids blocked more than 50% of the posterior nostrils or nasopharyngeal A/n ratio was 50% or more; 3) Surgical treatment is planned after 1 month; 4) The patient and his family had no history of montelukast allergy; 5) No surgical contraindications, the family members of the child signed the informed consent form. Exclusion criteria: 1) Children with adenoidal hypertrophy combined with tonsillar hypertrophy or who have undergone tonsillectomy; 2) Children with comorbidities, such as secretory otitis media, chronic sinusitis, etc; 3) Children with craniofacial deformities, such as cleft palate and choanal atresia; 4) Current or previous use of montelukast sodium, acute upper respiratory tract infection, use of any corticosteroids, or use of antibiotics within 4 weeks prior to the study; (5)Severe cardiopulmonary dysfunction; 6) With severe infection.

#### 1.1.3 Study design

20 patients were randomized into two groups: montelukast group and control group. Randomization is performed by a computer-generated table Ofrandom number: If the first digit of the random number was 0–4, the patient was assigned to the montelukast sodium group, and if the first digit was 5-9, the patient was assigned to the control group. The montelukast sodium group (n = 10) was given cysteine leukotriene receptor antagonist -montelukast sodium chewable tablets (Merck Sharp &Dohme B.V.), 5mg/time, once/day. And require all parents to take pills before the child goes to bed for 4 weeks. The minimum age of use of montelukast sodium was 2 years, and the children in the study were between 3 and 8 years of age. We use the recommended dose for the treatment of nasal symptoms of seasonal allergic rhinitis and perennial allergic rhinitis:5mg/time, once/day. The control group (n = 10) was prescribed placebo tablets with the same shape, color and dose: 5mg/time, once/day. Investigators contacted parents weekly to determine compliance and to follow up on potential side effects. Four weeks later, all patients had venous blood collected before surgery for blood cell analysis. Subsequently, all patients underwent plasma radiofrequency assisted adenoid ablation under general anesthesia. And adenoid tissue was collected during the procedure. Macroscopically, it was seen as a grayish-white tissue mass of about 0.3 × 0.4 cm in size. It was preserved in a formaldehyde tube, and the excised adenoid tissue was examined by light microscopy. Tissue specimens were fixed in 10% neutral buffered formalin, dehydrated with graded alcohol series, removed with xylene, and embedded in paraffin. 5μm thick paraffin sections were stained with rosewood essence and eosin and examined under light microscope. Each patient is magnified at high magnification (×40,×200) Check 10 slices below. The pathologist examined each section for inflammatory cell infiltrates, lymphoid follicular structures, and congested blood vessels. The findings of the two groups were compared. All slides were examined by the same pathologist and photographed.

#### 1.1.4 Statistical analysis

SPSS 25.0 was applied to the data analysis of this study. The Kolmogorov Smirnov test is used to judge the normality of the measurement data. If it conforms to the normal distribution, the mean value ±standard deviation is used to represent it. Otherwise, the median (quartile interval) is used to represent it. The measurement data between the two groups were analyzed by independent sample *t*-test or rank sum test, and the categorical variable was analyzed by chi square test.

## 2 Result

**FIGURE 1:**
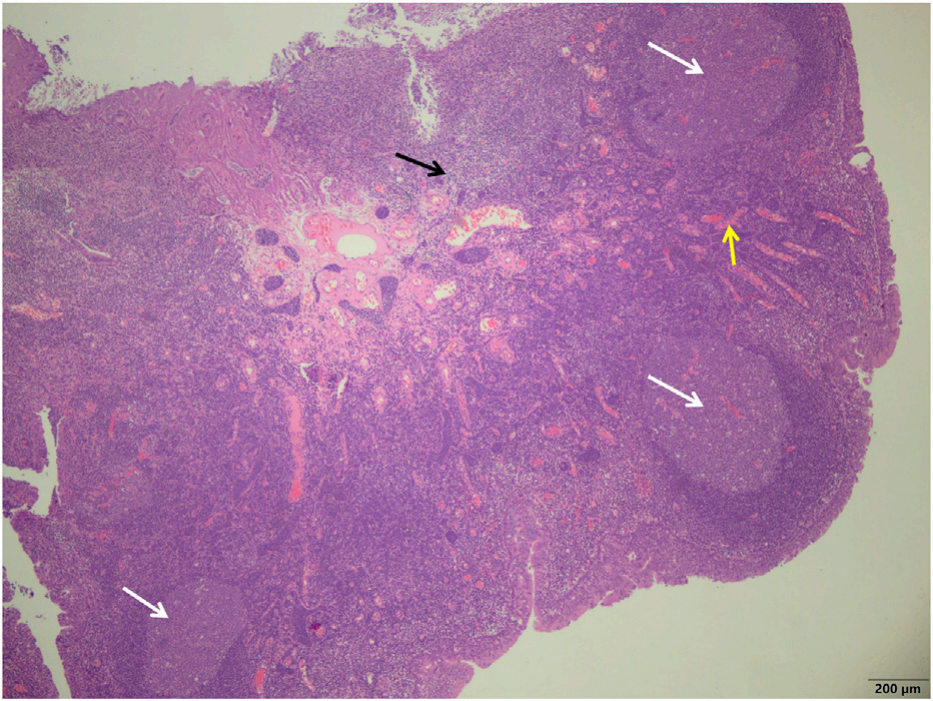
Study group showed a more uniform size of the germinal centers (white arrows), a smaller number of reactive germinal centers, and a significant reduction in the number of interstitial inflammatory cells (black arrows) and a reduction in interstitial congestion and hemorrhage (yellow arrows) (H&E; ×40).

**FIGURE 2:**
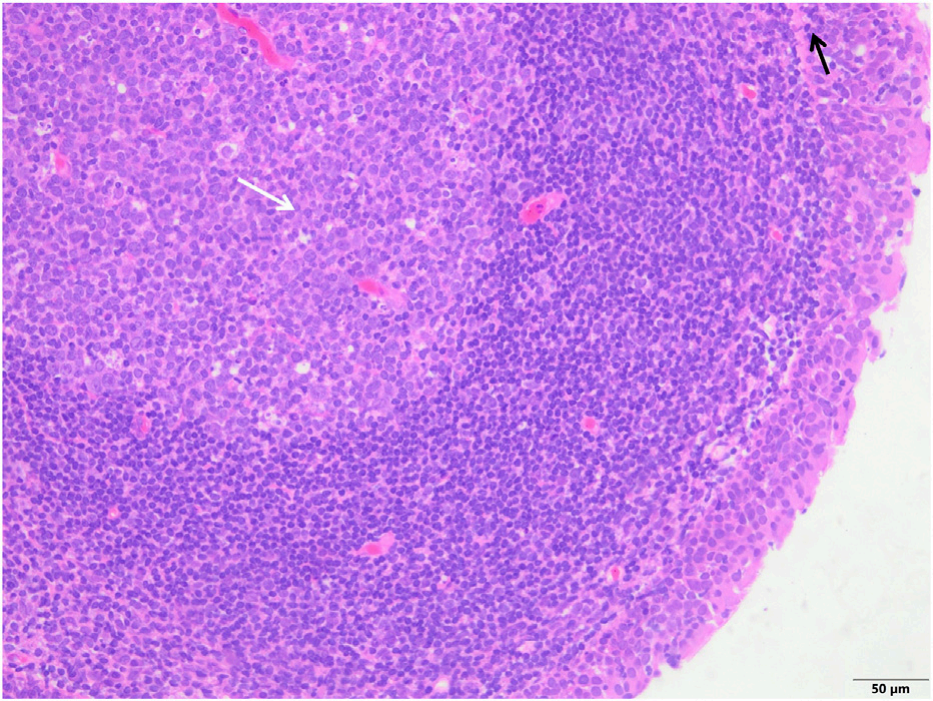
The study group showed that the epithelial structure was intact, no cell degeneration, necrosis, no bleeding in the interstitium, fewer inflammatory cells (black arrow), and a clear structure of the germinal center (white arrow) (H&E; ×200).

**FIGURE 3:**
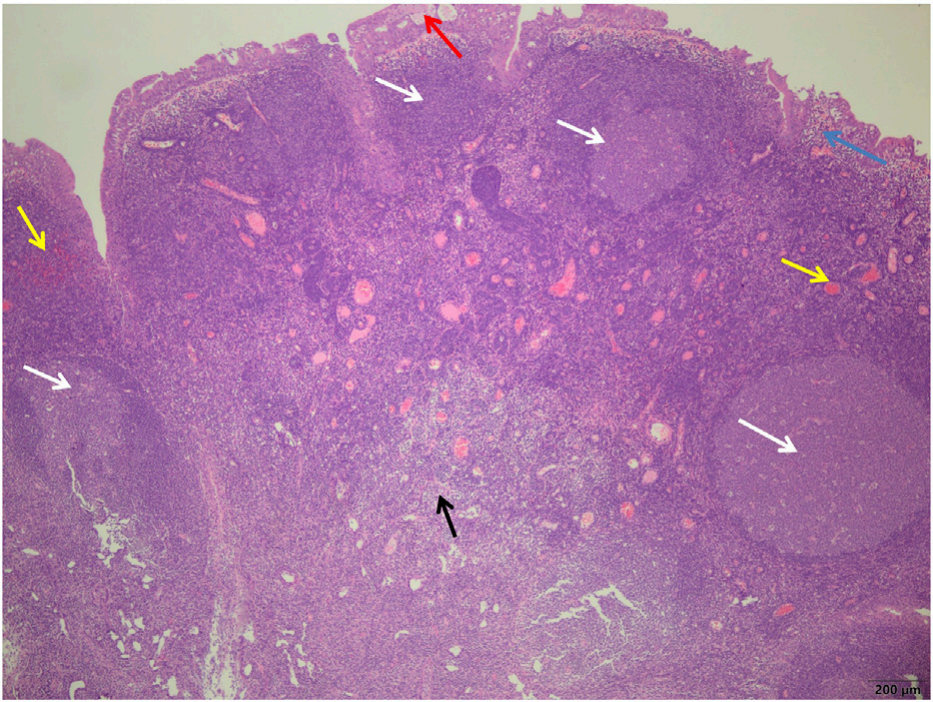
Control group showed more germinal centers (white arrows) surrounded by well-defined mantle bands, small cyst formation in the glandular epithelium (red arrows), subepithelial edema (blue arrows), more interstitial hyperemia, bleeding (yellow arrows), and more chronic inflammatory cell infiltration (black arrows) (H&E; ×40).

**FIGURE 4:**
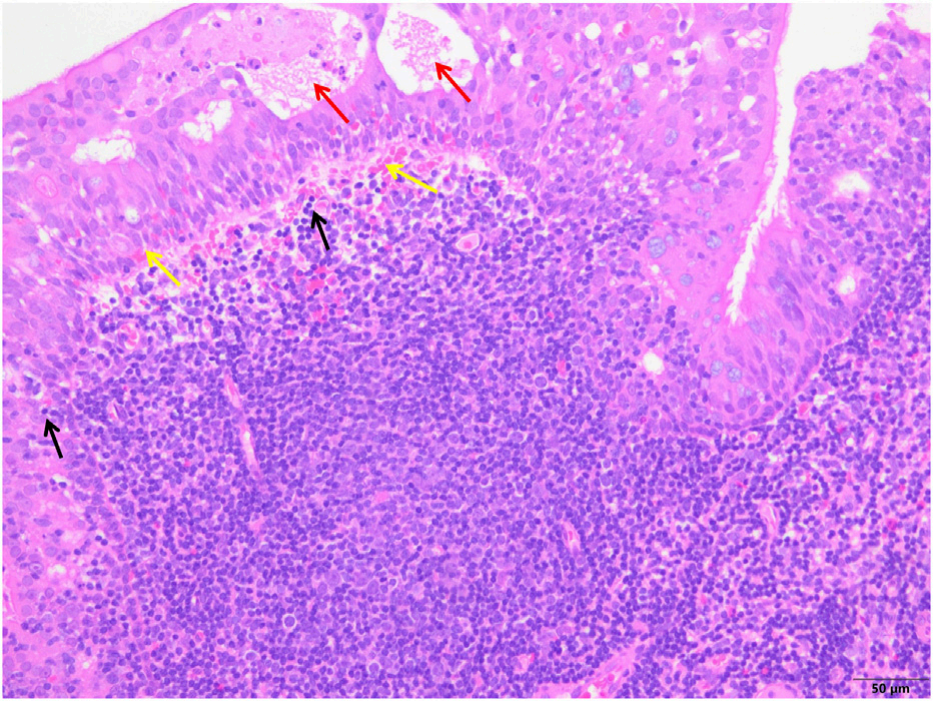
The control group showed epithelial degeneration and necrosis followed by the formation of small cysts (red arrows), more subepithelial hemorrhages (yellow arrows) and inflammatory cell infiltration (black arrows), and unclear structure of reactive germinal centers (white arrows) (H&E; ×200).

A total of 20 children (8 males and 12 females, 3–8 years old, mean 5.10 ± 1.48 years) were enrolled in our study. No patients withdrew and no side effects were reported. There was no significant difference in general conditions (age, sex, height, weight, BMI) between the two groups (*p* > 0.05) (Table 1). Compared with the control group, there was a significant decrease in lymphocytes in the blood cell analysis of the study group, with a statistically significant difference (*p* < 0.05) (Table2). The experimental group showed under the light microscope that the number of reactive germinal centers of adenoidal tissue was reduced and more uniform in size, no small cysts were seen in the epithelium, the degree of inflammatory cell infiltration was reduced, and the number of interstitial inflammatory cells was significantly reduced. (Figure 1 and Figure 2). In control group, proliferative and reactive lymphoid follicles with different sizes and shapes were observed, and the blood vessels in the interfollicular zone were congested. And small cysts are visible in the epithelium and the degree of inflammatory cell infiltration is severe (Figure 3 and Figure 4). The results of histopathological examination showed that compared with the control group, the number of adenoid tissue germination centers, the number of cystic cavities, and inflammatory cell infiltration in the experimental group were significantly reduced, and the differences were statistically significant (*p* < 0.05) (Table 3). This supports the efficacy of montelukast sodium in reducing the immune and inflammatory response in adenoidal tissue, as well as its potential to reduce its size.

**TABLE 1:** Demography and clinical characteristics of the experimental group and the control group.

Category	Group		*p*-value
	Experimental group	Control group	
Number	10	10	-
Age	5 (1.0)	4 (2.0)	0.294^✝^
Sex	4 (40.0)	5 (50.0)	0.653^a^
	6 (60.0)	5 (50.0)	
height	113.50 ± 8.62	111.20 ± 14.08	0.681^b^
weight	19.50 ± 3.26	19.80 ± 6.00	0.897^b^
BMI	15.05 ± 1.20	15.73 ± 1.87	0.373^b^

^a^ Chi-square test.

^✝^ Mannwhitney-U.

^b^ *t*-test.

**TABLE 2:** Blood cell analysis in the experimental and control groups.

Category	Group		*p*-value
	Experimental group	Control group	
Leukocyte	6.73 (1.41)	6.85 (1.65)	0.273^✝^
Basophil	3.60 ± 2.20	5.30 ± 2.33	0.129^a^
Eosinophils	2.50 (0.60)	2.60 (1.60)	0.596^✝^
Monocyte	5.90 (1.10)	6.20 (0.50)	0.306^✝^
Lymphocyte	48.30 (12.50)	41.20 (2.70)	0.031^✝^
Neutrophils	38.70 (17.70)	48.30 (4.40)	0.104^✝^

Categorical variables were measured in 10 *^9^.

Chi-square test.

^✝^ Mannwhitney-U.

^a^ *t*-test.

**TABLE 3:** Histopathological examination results of experimental and control groups.

Category	Group		*p*-value
	Experimental group	Control group	
Number of germinal centers	8.70 ± 7.56	16.50 ± 6.36	0.029^a^
Cystic cavities	0.00 ± 0.00	0.60 ± 0.66	0.024^a^
Inflammatory cell infiltration	1.10 ± 0.94	2.00 ± 0.77	0.040^a^

^a^ *t*-test.

## 3 Discussion

After 4 weeks of oral administration of montelukast sodium, histopathological evaluation of adenoid tissue showed that compared with the control group, the number of germinal centers in adenoid tissue in the study group was relatively reduced, no small cyst was found in the epithelium, and the degree of inflammatory cell infiltration was reduced. Compared with the control group, lymphocytes were significantly reduced in blood cell analysis in the study group. The control group showed proliferative, reactive, lymphatic follicles of different sizes and shapes, vascular congestion in the interfollicular region, small cysts in the epithelium, and severe infiltration of inflammatory cells. This supports the potential of oral montelukast sodium tablets to reduce immune and inflammatory responses in adenoidal tissue and to reduce their size.

The adenoids are lymphoepithelial tissues that form part of the Waldeyer ring and form part of the mucosal immune system (Fossum et al., 2017). Its main function is to participate in the secondary immune system by immunologically sampling antigens and local pathogens (Arambula et al., 2021). Histologically, adenoids are mainly composed of pseudostratified ciliated columnar epithelium, with lymphatic follicles arranged in mucosal folds. The connective tissue septum extends from the capsule to the pharyngeal tonsil tissue, dividing it into 4-6 segments (Goldwyn, 1985; Brandtzaeg, 2003). Exogenous antigens are absorbed through the nose/oropharynx, processed by antigen-presenting cells (APCs), and then presented to T and B-cell in the adjacent follicular outer zone. If an antigen has been encountered before, a secondary immune response is generated through T-cell proliferation and/or B-cell (Nave et al., 2001). If the antigen encountered is novel and successfully recognized by the helper T-cell. The T-cell population specific for that antigen is activated, proliferated, and differentiated as long as appropriate costimulatory signals are present. These T-cell stimulate primary B-cell, which then reach nearby follicles and differentiate into antigen-specific plasma cells and memory B-cell, eventually forming germinal centers (van Kempen et al., 2000). The greater the antigenic stimulation, the more active the germinal centers and the larger the adenoid tissue (Scadding, 1990).

Bernstein’s studies have shown that adenoidal hypertrophy is caused by local and systemic immune dysfunction (Bernstein et al., 1993). The number of lymphocytes in adenoids and their role in immune response depend on their proliferation and migration status, and apoptosis provides a balance between lymphocytes. The increase or decrease in cell apoptosis determines the progression of the inflammatory process. Reduced cell apoptosis leads to increased severity of chronic inflammation and diseases (Önal et al., 2015). In normal lymphoid tissue, cell apoptosis and proliferation must be balanced to ensure the stability of the total number of lymphocytes (Rathmell and Thompson, 2002). In the adenoid tissue of patients with adenoid hypertrophy, there is an imbalance in the regulation of cell proliferation and apoptosis, with cell proliferation dominating. Adenoid hypertrophy is secondary to lymphoid hyperplasia. Li,Ke-Xin et al. pointed out that leukotrienes have a certain anti-apoptosis effect (Li et al., 2020). Meanwhile, other studies have shown that LTD4 plays a role in the proliferation of CD4 + T-cell and CD8 + T-cell through MAPK signaling pathway (Kaditis et al., 2008). Montelukast, as a leukotriene receptor antagonist, can play the opposite role, promoting apoptosis and inhibiting proliferation, thereby reducing hypertrophy of adenoids.

Montelukast sodium is an orally bioavailable cysteine leukotriene (LT) receptor antagonist with good efficacy, high safety, and no significant tolerability (Storms et al., 2001), which can be used for the prevention and treatment of asthma and allergic rhinitis in children over 2 years of age (Knorr et al., 2001). Leukotrienes are key inflammatory mediators in the respiratory system. These lipid mediators are involved in the pathogenesis of childhood diseases such as asthma. They are also systemically and locally involved in the inflammatory process in children with AH (4). Studies have confirmed an increased expression level of LT receptors in the upper lymphoid tissue of obstructive sleep apnea patients (Goldbart et al., 2004). Moreover, the study by David Gozal et al. further confirmed that the concentration of LT in adenoidal tissue also increased, which indicated that there was an active inflammatory process in the upper respiratory tract of children, and the synergistic increase of LT production and receptor expression might be the basis of the signaling pathway leading to proliferation and hyperplasia of lymphoid tissue in these children (Goldbart et al., 2005). Somers VK et al. also demonstrated elevated levels of c-reactive protein in hypertrophic adenoidal tissue, suggesting a local chronic inflammatory process associated with proliferation of adenoid lymphoid tissue (Shamsuzzaman et al., 2002). Goldbart observed high levels of cysteinyl leukotriene (CysLTs) and high expression of CysLTs receptor in adenoids and tonsil tissues of children with OSA in 2005 (Goldbart et al., 2005). In the past few years, researchers have also repeatedly demonstrated that cyslt-mediated allergic reactions are associated with abnormal hyperplasia of adenoidal and tonsillar hypertrophy in children with OSA (Tsaoussoglou et al., 2014; Feng et al., 2015; Mousailidis et al., 2018). Montelukast is a typical leukotriene receptor antagonist, which is commonly used to control inflammatory response (Nachalon et al., 2014). Based on these theories, we have reason to believe that montelukast sodium can control the development of inflammation in adenoidal tissue in children with adenoidal hypertrophy (Ji et al., 2021).

Macroscopically, studies have shown that regular oral administration of montelukast is effective in reducing adenoid volume. Farshid Shokouhi et al. showed that Montelukast oral chewable tablets can reduce the size of glandular tissue in addition to effectively relieving snoring, sleep discomfort and the severity of oral breathing after 12 weeks of treatment in children with AH (4). Goldbart further found that montelukast significantly reduced adenoidal volume and improved upper airway patency (Goldbart et al., 2005). And a study by Goldbart et al., 2012 showed the use of montelukast to treat OSA symptoms in 40 children aged 4–12 years (Goldbart et al., 2012). 20 children received Montelukast chewable tablets (4 mg for children <6 years old, 5 mg for older children), while another 20 children received placebo. After 12 weeks, the parameters of polysomnography improved significantly (>50%), and the X-ray A/N ratio decreased from 81% to 57%. They recommend this treatment for mild OSA. Liming et al. systematically reviewed six studies (one prospective cross-sectional interview), two prospective cohorts, one retrospective cohort, and two placebo controlled prospective randomized controlled trials) were conducted to investigate the use of anti-inflammatory drugs in the treatment of obstructive sleep apnea (OSA) in children (Liming et al., 2019). In five studies, it was found that montelukast alone improved AHI and minimal SpO2 compared to before treatment. We have reason to believe that montelukast sodium can reduce the volume of adenoids, increase ventilation volume, and alleviate symptoms in patients.

According to studies, montelukast sodium can significantly improve the clinical symptoms of children with OA, the polysomnography (PSG) monitoring parameters, and the SDB-related questionnaire scores (Ji et al., 2021). But the side effects of montelukast sodium are also a problem that we should pay attention to. In addition to common adverse reactions such as headache, nausea and vomiting, allergy, and fever (Goldbart et al., 2012), the potential risk of its associated neuropsychiatric adverse reaction cannot be excluded (Callero-Viera et al., 2012). Therefore, clinicians must pay attention to the safety of montelukast and strictly control the indications and contraindications in adults and children. They should also control the dosage of use and ask detailed questions about past medical history and psychiatric history to avoid the occurrence of adverse reactions.

Our study reported histopathology changes under the light microscope of montelukast sodium for the first time, but did not carry out immunohistochemistry, which will be possible to better identify the cell and structural changes in adenoid tissue. A month of treatment with this dose of montelukast sodium is not enough to eliminate enlarged adenoids that require surgery. Further research with a longer treatment cycle and different doses is needed to determine whether surgical treatment for children with small hypertrophic adenoids can be avoided.

## 4 Conclusion

Four weeks after oral administration of montelukast sodium, the histopathology evaluation of adenoids showed that the number of germinal centers in the study group was relatively reduced, no small cysts were found in the epithelium, and the degree of inflammatory cell infiltration was reduced. Compared with the control group, lymphocytes were significantly reduced in blood cell analysis in the study group. This supports the potential of oral montelukast sodium tablets in reducing immune and inflammatory responses in adenoid tissue and reducing its size. However, this is a preliminary study, and further studies with a longer treatment period and different doses are needed to evaluate the effect of montelukast sodium on the size of adenoids and histopathology, and explore the possibility of its use in perioperative preparation or alternative non-invasive surgery.

## Data Availability

The original contributions presented in the study are included in the article/Supplementary Materials, further inquiries can be directed to the corresponding author.
