# Supplementary material for: Effects of oral cysteine leukotriene receptor antagonist-montelukast on adenoid lymphoid tissue: a histopathological study under light microscope
Source: Front Pharmacol. 2023 Nov 2;14:1285647. doi: 10.3389/fphar.2023.1285647 (PMC10651758; doi:10.3389/fphar.2023.1285647)
Supplement: Supplementary file 1 [file Table1.DOCX]

**Table1** Demography and clinical characteristics of the experimental group and the control group

| Category | | Group | | | | *p*-value | |
| --- | --- | --- | --- | --- | --- | --- | --- |
|  |  | experimental group | | control group | |  |  |
| Number | 10 | | 10 | | - | |  |
| Age | 5(1.0) | | 4(2.0) | | 0.294^✝^ | |  |
| Sex | 4(40.0) | | 5(50.0) | | 0.653^*^ | |  |
|  | 6(60.0) | | 5(50.0) | |  |  |  |
| height | 113.50±8.62 | | 111.20±14.08 | | 0.681^#^ | |  |
| weight | 19.50±3.26 | | 19.80±6.00 | | 0.897^#^ | |  |
| BMI | 15.05±1.20 | | 15.73±1.87 | | 0.373^#^ | |  |

^*^Chi-square test ^✝^ Mannwhitney-U ^#^ t-test

**Table2** Blood cell analysis in the experimental and control groups

| Category | | Group | | | | *p*-value | |
| --- | --- | --- | --- | --- | --- | --- | --- |
|  |  | experimental group | | control group | |  |  |
| Leukocyte | 6.73（1.41） | | 6.85(1.65) | | 0.273^✝^ | |  |
| Basophil | 3.60±2.20 | | 5.30±2.33 | | 0.129^#^ | |  |
| Eosinophils | 2.50(0.60) | | 2.60(1.60) | | 0.596^✝^ | |  |
| Monocyte | 5.90（1.10） | | 6.20（0.50） | | 0.306^✝^ | |  |
| Lymphocyte | 48.30（12.50） | | 41.20（2.70） | | 0.031^✝^ | |  |
| Neutrophils | 38.70（17.70） | | 48.30（4.40） | | 0.104^✝^ | |  |

Categorical variables were measured in 10 * ^9^

Chi-square test ^✝^ Mannwhitney-U ^#^ t-test

**Table3** Histopathological examination results of experimental and control groups

| Category | Group | | | *p*-value | |
| --- | --- | --- | --- | --- | --- |
|  | experimental group | | control group |  |  |
| Number of germinal centers | 8.70±7.56 | 16.50±6.36 | | 0.029^#^ |  |
| Cystic cavities | 0.00±0.00 | 0.60±0.66 | | 0.024^#^ |  |
| Inflammatory cell infiltration | 1.10±0.94 | 2.00±0.77 | | 0.040^#^ |  |

^#^ t-test
